# Cache Pilfering Risks Vary With Seed Species and Pilferers

**DOI:** 10.1002/ece3.72337

**Published:** 2025-10-16

**Authors:** Muha Cha, Yuan Li, Minghui Wang, Xianfeng Yi

**Affiliations:** ^1^ Academy of Agricultural Sciences Chifeng University Chifeng China; ^2^ Enshi Tujia and Miao Autonomous Prefecture Academy of Forestry Enshi China; ^3^ School of Life Sciences Qufu Normal University Qufu China

**Keywords:** cache pilfering risk, re‐caching, rodent identity, scatter‐hoarding, seed trait, synzoochory

## Abstract

Cache pilfering is a pervasive phenomenon among seed‐hoarding rodent species; nonetheless, the influence of seed species and pilferer identity on cache pilfering risk at the community level remains underexplored. This study examined the cache pilfering risk of the scatter‐hoarding rodent *Leopoldamys edwardsi* and investigated the subsequent fate of the pilfered seeds of two dominant tree species, *Camellia oleifera* and *Castanopsis henryi*. The experiments were conducted in semi‐natural enclosures, which simulate natural habitat conditions while allowing for controlled observation of rodent behavior. Sympatric pilfering rodent species involved in the study also comprised *Apodemus chevrieri* , *A. draco* , *Niviventer confucianus* , and *N. fulvescens* , all commonly found in the study area. Our findings revealed that *L. edwardsi* showed no significant preference between the seeds of *C. henryi* and *C. oleifera* , but the pilferage rate of *C. oleifera* seeds was significantly higher. The cache pilfering risk posed by sympatric rodent species varied significantly, with scatter‐hoarding pilferers being primarily responsible for most cache losses. Moreover, seed species exerted a substantial impact on the cache pilfering risk imposed by these pilferers. Notably, we discovered that scatter‐hoarding pilferers selectively re‐cached pilfered seeds on the basis of seed species, a process that is anticipated to play a pivotal role in secondary seed dispersal and, consequently, plant regeneration.

## Graphical abstract

Our findings revealed that the cache pilfering risk posed by sympatric rodent species varied significantly, with scatter‐hoarding pilferers being primarily responsible for most cache losses. Moreover, seed species exerted a substantial impact on the cache pilfering risk imposed by these pilferers.

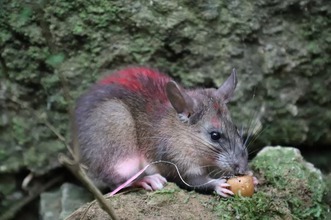

## 1 Introduction

Rodents encountering seeds typically have three possible responses: rejection, consumption, or storage (Shimada 2001; Lai et al. 2025). Food hoarding is indispensable for rodents to endure periods of food scarcity (Zhang et al. 2022; Gan et al. 2024). Broadly speaking, rodents employ two predominant food‐caching strategies: scatter‐hoarding and larder‐hoarding (Niu et al. 2020; Deng et al. 2020). Scatter‐hoarding involves the creation of numerous small, unguarded caches dispersed throughout an animal's home range (Cheng et al. 2025). In contrast, larder‐hoarding animals consolidate their collected food items into a single, often well‐defended location, such as a rock fissure or burrow chamber within a confined area (Dittel et al. 2017; Wang and Yi 2022). Although both types of hoards are susceptible to pilferage by sympatric competitors, cache pilfering disproportionately affects scatter‐hoarders (Vander Wall and Jenkins 2003; Steele et al. 2011; Jansen et al. 2012). Seeds cached via scatter‐hoarding frequently face elevated risks of pilferage from both inter‐ and intraspecific rodent competitors (Lichti et al. 2017; Wang et al. 2018), as larder‐hoarded seeds are typically fiercely guarded by their owners. Because of the highly pervasive and persistent threat of pilferage that is posed by both interspecific and intraspecific rivals, hoarding animals have adopted a strategic approach to mitigate their potential food losses. Reciprocal pilfering refers to the mutual stealing or taking of seeds among different organisms or species, having significant implications for the process of seed secondary dispersal. This complex interaction pattern thereby significantly underscores the truly pivotal role that interspecific pilfering plays in shaping the competitive dynamics at the community level. The implications of such behavior extend far beyond individual survival, influencing the overall balance and stability within the entire community ecosystem (Dittel et al. 2017). Therefore, understanding the complex relationship between reciprocal pilfering and these ecological factors is essential for comprehensive ecological research and conservation efforts.

Food hoarding behaviors typically exhibit interspecific variation among sympatric rodent species (Meng et al. 2023; Yu et al. 2023). Some species may exclusively engage in scatter‐hoarding or larder‐hoarding, whereas others display a dual strategy by combining both behaviors (Wang et al. 2014; Zhang et al. 2016; Geng et al. 2017). Interspecific differences in olfactory abilities have been documented among granivorous rodents (Vander Wall et al. 2003). Scatter‐hoarders tend to be more adept at pilfering compared to larder‐hoarders, likely because of their heightened olfactory sensitivity (Wang et al. 2018). Cache pilferers, who are often opportunistic creatures, predominantly rely on their acute sense of olfaction to precisely locate the buried seeds (Vander Wall et al. 2005). This is because they lack the remarkable spatial memory that the original hoarders possess. The original hoarders are the individuals who initially cache the seeds by burying them for future use, whereas cache pilferers lack the remarkable spatial memory that the original hoarders possess (Wang et al. 2018). As a result, the pilferers have to depend mainly on their sense of smell to detect and uncover the hidden treasures of seeds (Yi et al. 2016). Consequently, caches are at a greater risk of pilferage when scatter‐hoarders, rather than larder‐hoarders, participate in theft. Furthermore, seed traits significantly influence the predation, hoarding, and pilfering behaviors of rodents (Cao et al. 2016; Zhang et al. 2016; Chen et al. 2022; Xiao et al. 2022). The intensity of seed odor emitted from caches has been shown to dictate the pilfering risk posed by rodent theft (Hollander et al. 2012; Dimitri and Longland 2022). However, rodents exhibit pronounced preferences for certain odors when foraging for food items (Jagetia et al. 2018). Thus, the pilfering risk of seed caches is anticipated to vary across seed species, depending on whether the pilfering is conducted by scatter‐hoarders or larder‐hoarders.

In the Dujiangyan region of China, a diverse community of rodents coexists and competes for limited food resources. Although cache pilfering is prevalent among seed‐hoarding rodent species (Yi et al. 2016; Niu et al. 2020; Wang et al. 2023), the influence of seed species on cache pilfering risk at the community level remains underexplored. Furthermore, previous studies delving into the phenomenon of cache pilferage have frequently failed to take into account the successive re‐caching process of excavated seeds (Hollander et al. 2012; Wang et al. 2025). Wang et al. (2019) considered re‐caching behavior and demonstrated its role in enhancing seed dispersal effectiveness. This oversight is significant as it may give rise to profound and far‐reaching ecological consequences. For instance, it could potentially enhance the distances over which seeds are dispersed, allowing them to reach new and previously uncolonized areas (Jansen et al. 2002; Perea et al. 2011; Hirsch et al. 2012). Additionally, it might improve the survival probabilities of the seeds, providing them with better chances to germinate and establish new plant populations. Such effects could have a substantial impact on the overall structure and dynamics of forest and plant communities. A detailed examination of re‐caching processes could reveal complex interrelationships and dependencies within the ecosystem, highlighting the importance of understanding seed caching and its associated behaviors for effective conservation and management strategies.

In this study, we explored the cache pilfering risk of the scatter‐hoarding rodent *Leopoldamys edwardsi* within semi‐natural enclosures, considering interactions mediated by sympatric rodent species: *Apodemus chevrieri* , *A. draco* , *Niviventer confucianus* , and *N. fulvescens* . This community‐level approach reflects the combined effects of multiple co‐occurring species on cache pilferage. Semi‐natural enclosure experiments have been widely used in seed cache studies to balance ecological relevance with controlled observation, allowing researchers to investigate animal behavior and species interactions under conditions that approximate natural environments. To examine whether seed species influence cache pilfering and the subsequent re‐caching behavior of pilferers, we employed two seed species (*Castanea henryi* and *Camellia oleifera*) that exhibit markedly distinct seed traits. On the basis of prior knowledge, we hypothesize: (1) Cache pilfering risk imposed by scatter‐hoarders will exceed that of larder‐hoarders, as scatter‐hoarders possess superior pilfering abilities; (2) Seed species will interact with pilferer identity to modulate cache pilfering risk, driven by variations in seed odor emitted from caches; and (3) The successive re‐caching behavior of pilferers will be contingent upon seed species.

## 2 Materials and Methods

### 2.1 Study Site

This study was conducted in Dujiangyan City, Sichuan Province, China (31°4′ N, 103°43′ E) from October 2018 to January 2019. The study area is characterized by a subtropical evergreen zone, where the climate is predominantly cloudy and foggy (Chang and Zhang 2011). The local relative humidity typically exceeds 80%, with an annual sunshine duration of 800–1000 h, an average annual temperature of 15.2°C, and an average annual rainfall ranging from 1200 to 1800 mm (Yang et al. 2019).

### 2.2 Rodent Species

In this region, Edward's long‐tailed rats ( *L. edwardsi* ), Chevrier's field mice ( *A. chevrieri* ), South China field mice ( *A. draco* ), Chinese white‐bellied rats ( *N. confucianus* ), and chestnut rats ( *N. fulvescens* ) are the primary seed dispersers (Yang et al. 2019). Among these species, *L. edwardsi* dominates as scatter‐hoarders, whereas *A. chevrieri* and *A. draco* primarily engage in larder‐hoarding but occasionally exhibit scatter‐hoarding behavior. In contrast, *N. confucianus* and *N. fulvescens* exclusively practice larder‐hoarding (Wang et al. 2018; Wang and Yi 2022).

### 2.3 Trapping Methods

To capture these animals, we employed live‐wire traps (30 cm × 13 cm × 12 cm) pre‐baited with peanuts and carrots. The traps were set between 18:00 and 19:00 and inspected at 8:00 the following morning. Captured animals were transported to our laboratory for housing, with the exception of pregnant or juvenile individuals, who were immediately released at the capture site. All animals were housed individually in spacious mouse cages (45 cm × 30 cm × 15 cm) equipped with nesting material. They were provided ad libitum access to rat chow (4% fat, 20% protein, 70% carbohydrate; Shenyang Maohua Biotechnology Co. Ltd., Liaoning, China) and fresh drinking water. The housing environment was maintained at a temperature of 20°C–25°C under a natural light/dark cycle (12L:12D). Following the completion of the experiments, all animals were returned to their original capture sites.

### 2.4 Seed Species

In the autumn of 2018, we meticulously gathered fresh seeds of *C. henryi* and *C. oleifera* directly from the forest floor. Seeds of uniform size for each species were carefully selected using a floating water method to identify and exclude those damaged by insects or otherwise rendered hollow. We chose these two tree species because of their pronounced differences in both physical and chemical characteristics. The fresh seed mass of *C. henryi* is notably larger than that of *C. oleifera* (Table 1). The crude fat content is significantly higher in *C. oleifera* seeds compared to *C. henryi* seeds, whereas the crude starch content exhibits the opposite trend. No significant differences were observed in crude fiber, crude protein, or caloric value between the two species (Xiao et al. 2008; Yang et al. 2019). Seeds of *C. oleifera* may have a stronger odor emission than those of *C. henryi* because *C. oleifera* are easy to detect (Wang and Yi 2022). To facilitate tracking, a 1.5 mm diameter hole was drilled into each seed, and a Passive Integrated Transponder (PIT) tag (model: RBC‐1.4*8B‐B‐J; dimensions: 1.4 mm × 8 mm; weight: 0.03 g) was securely inserted into the hole. We ensured that the PIT tags would remain firmly in place during handling by rodents, preventing any potential detachment. During the drilling and tag implantation procedures, we wore plastic gloves to avoid any possible interference from human odors with the rodents' search behavior or the provision of inadvertent cues. The probability of tags being inadvertently ingested by rodents is relatively low (Roberts et al. 2021). Moreover, if ingestion occurs, the tags can be effectively expelled from the body through the digestive system without adverse effects. As such, these tags do not constitute a significant risk to rodent populations.

**TABLE 1:** Comparison of seed traits.

	Fresh weight (g)	Crude fat content (%)	Crude starch content (%)	Crude fiber content (%)	Crude protein content (%)	Energy value (kJ)	Odor intensity
*C. henryi*	6.25 ± 0.87	1.11	58.71	2.31	7.05	16.58	Weaker
*C. oleifera*	1.50 ± 0.35	52.92	1.87	3.32	8.63	22.96	Stronger

### 2.5 Enclosure Design

**FIGURE 1:**
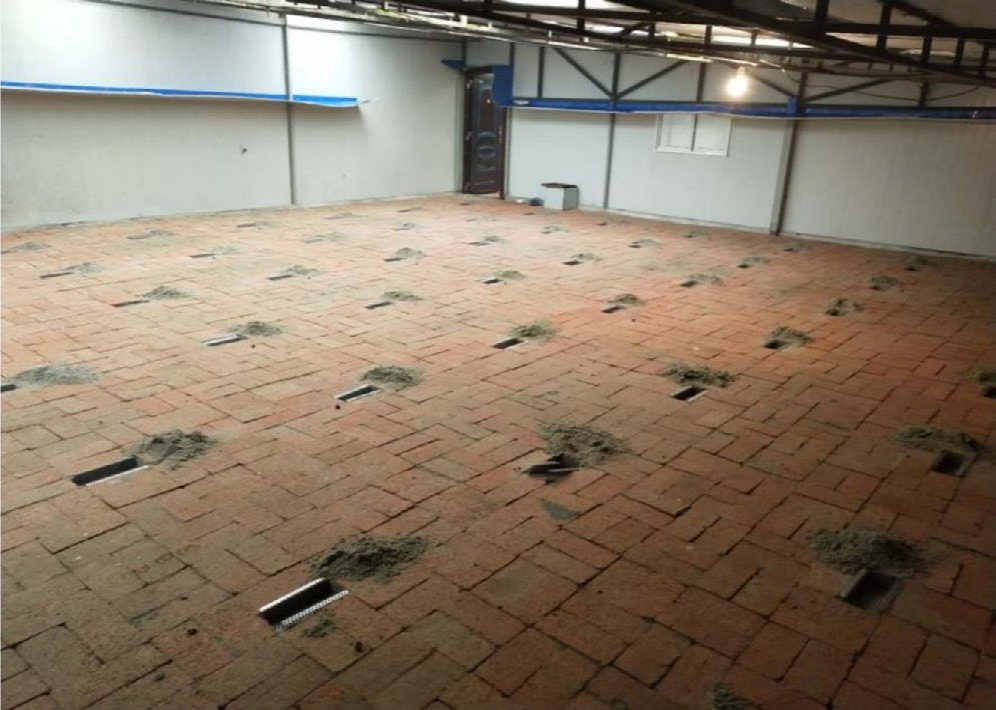
Schematic diagram of the enclosure design.

This study was conducted in five semi‐natural enclosures (Figure 1), each measuring 10 m × 10 m × 2.0 m, established adjacent to the forested areas. The enclosures were constructed with bricks, featuring smooth walls that extended 2.0 m above ground and 0.5 m below to prevent escape or intrusion. A transparent roof supported by a steel frame covered the top of the enclosures, ensuring protection from predators while maintaining natural light conditions. The floor of each enclosure was paved with red bricks, creating 64 evenly distributed shallow pits (24 cm × 12 cm × 6 cm), each filled with fine sand to facilitate seed caching by rodents. Two nesting boxes (40 cm × 40 cm × 40 cm) and two water plates were strategically placed in the corners of each enclosure to provide warmth for experimental animals and ensure an adequate supply of drinking water. All animals were acclimatized within the enclosures for at least two nights before the behavioral experiments. We performed our experiments in semi‐natural enclosures mainly because it is hard to determine the cache pilfering behavior of focal animals in natural conditions (Wang et al. 2025).

### 2.6 Seed Hoarding and Cache Pilfering of *C. henryi* and *C. oleifera*

To evaluate the effects of seed species and pilferer identity on cache pilfering risk, we introduced individual *L. edwardsi* into the enclosures to establish caches, followed by sympatric rodent species to pilfer the cached seeds. After acclimatization, 40 labeled seeds of either *C. henryi* or *C. oleifera* were placed at the center of each enclosure. At 18:00, one individual *L. edwardsi* was introduced into each enclosure and allowed to move and forage freely for 13 h. By 07:00 the next morning, the hoarding animals were removed and confined to corresponding cages. Subsequently, the entire enclosure floor, including all shallow pits, was meticulously scanned using a handheld S03 PIT tag reader (Model RBC‐S03, Anhui Raybaca IoT Technology Limited Liability Company, Hefei City, Anhui Province) to locate intact seeds and seed debris. Seed fates were categorized into six groups: IIS (intact in situ), EIS (eaten in situ), IAR (intact after removal from the seed station), EAR (eaten after removal from the seed station), SH (scatter‐hoarded in shallow pits), and LH (larder‐hoarded in nesting boxes). Scatter‐hoarded seeds in the shallow pits were left undisturbed, whereas other intact seeds and seed debris were collected and removed from the enclosures. Although it would be more ecologically accurate to leave them in situ, these leftovers may not allow us to evaluate cache pilferage. At 18:00, another naïve animal was introduced into each enclosure and allowed to detect and excavate the cached seeds for 14 h. By 07:00 the following morning, the naïve animals were removed. The shallow pits were then carefully scanned again with the S03 PIT tag reader to determine how many seeds scatter‐hoarded by *L. edwardsi* were pilfered. When the cached seeds were pilfered, we also determined whether they were abandoned (S‐IAR), eaten at the cache site (S‐EIS), eaten far from the cache site (S‐EAR), re‐scatter‐hoarded (S‐SH), or re‐larder‐hoarded (S‐LH).

A total of 17 *L. edwardsi individuals* (9 males and 8 females; body weight: 470.6 ± 70.9 g, mean ± SD) were utilized to cache seeds of *C. henryi* . Subsequently, the following rodent species were individually introduced to pilfer these caches: 12 *L. edwardsi* (5 males and 7 females; 452.21 ± 70.83 g), 15 *A. chevrieri* (9 males and 6 females; 37.23 ± 4.00 g), 9 *A. draco* (5 males and 4 females; 22.63 ± 1.86 g), 14 *N. confucianus* (7 males and 7 females; 62.84 ± 10.00 g), and 15 *N. fulvescens* (8 males and 7 females; 67.52 ± 20.17 g). Similarly, 15 *L. edwardsi* individuals (9 males and 6 females; 448.7 ± 77.3 g) were employed to cache seeds of *C. oleifera* , followed by pilfering trials involving 10 *L. edwardsi* (6 males and 4 females; 511.0 ± 48.6 g), 12 *A. chevrieri* (10 males and 2 females; 38.06 ± 4.69 g), 5 *A. draco* (2 males and 3 females; 22.60 ± 2.01 g), 11 *N. confucianus* (6 males and 5 females; 64.41 ± 8.82 g), and 10 *N. fulvescens* (6 males and 4 females; 64.10 ± 17.42 g). Notably, although hoarding individuals may have been reused across trials, pilfering individuals were not employed repeatedly within this study.

### 2.7 Statistical Analyzes

All statistical analyses were performed using SPSS (version 25). Generalized linear models (GLMs) were constructed to assess the effect of seed species (*C. henryi* and *C. oleifera* ) on seed fate manipulated by *L. edwardsi* , with seed species as a fixed effect and seed fate as the response variable. On the basis of GLM models, the interaction effects of seed species and rodent species on cache pilferage rate were analyzed, with pilferage rate as the response variable and seed species, rodent species, and their interaction as fixed effects. Finally, to analyze the final fate of pilfered seeds, a GLM was established with seed species and rodent species as fixed effects. All models employed a binomial error distribution and logit link function. Model fit was evaluated using residual diagnostics and deviance goodness‐of‐fit tests to ensure that model assumptions were satisfied. Pairwise differences were tested using post hoc multiple comparisons with Bonferroni correction. Graphical figures were generated using GraphPad Prism (version 9.3). Data are presented as mean ± standard error of the mean (SEM), with individual data points overlaid where appropriate.

## 3 Results

### 3.1 Cache Pilfering Rate of *C. henryi* and *C. oleifera* by Rodent Species

**FIGURE 2:**
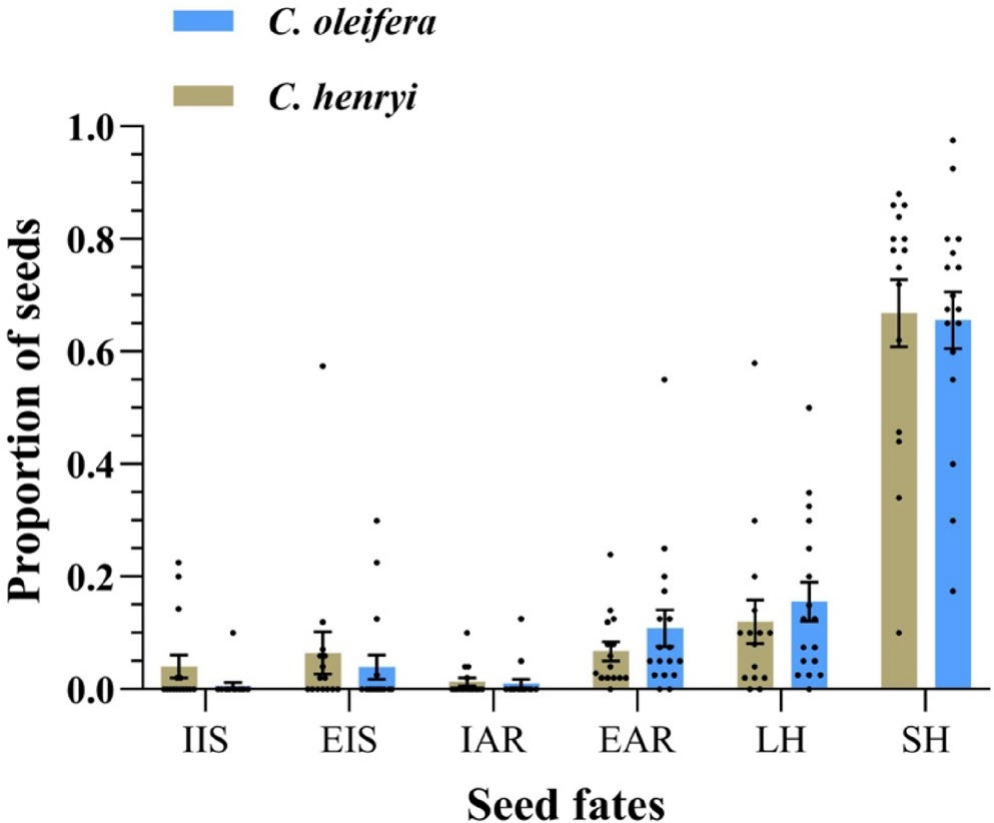
Seed fate (mean ± SE) of *Castanea henryi* and *Camellia oleifera* as manipulated by *Leopoldamys edwardsi* . IIS (intact in situ), EIS (eaten in situ), IAR (intact after removal), EAR (eaten after removal), LH (larder‐hoarded in the nesting boxes), and SH (scatter‐hoarded in the shallow pits).

*L. edwardsi* exhibited no significant preference between the seeds of *C. henryi* and *C. oleifera* , as indicated by the distribution of seed fates, including those left intact in situ, eaten in situ, eaten after removal, larder‐hoarded, or scatter‐hoarded (*χ* ^2^ = 3.168, df = 1, *p* > 0.05; *χ* ^2^ = 0.364, df = 1, *p* > 0.05; *χ* ^2^ = 1.245, df = 1, *p* > 0.05; *χ* ^2^ = 0.506, df = 1, *p* > 0.05; *χ* ^2^ = 0.028, df = 1, *p* > 0.05) (Figure 2).

**FIGURE 3:**
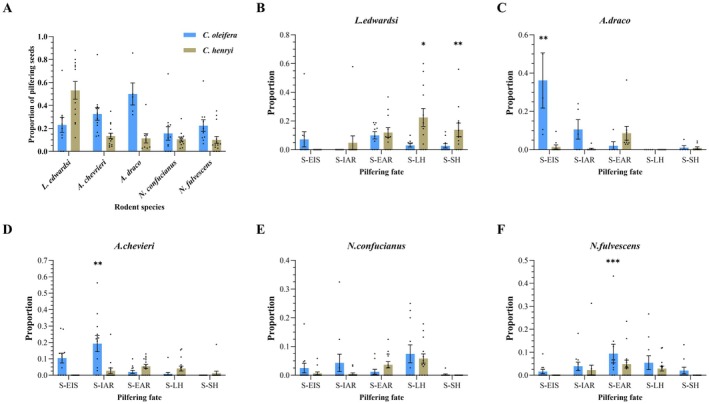
Cache pilfering risks (A) and successive fates of pilfered seeds (B–F) by sympatric rodent species. S‐EIS (eaten directly at the caches), S‐IAR (intact after excavation), S‐EAR (excavated and eaten away from the caches), S‐SH (excavated and re‐scatter‐hoarded in the shallow pits), and S‐LH (excavated and re‐larder‐hoarded in the nesting boxes). Asterisks (*, **, ***) denote significant differences between the two seed species at levels of *p* < 0.05, *p* < 0.01, and *p* < 0.001, respectively.

The results demonstrated that seeds of *C. oleifera* were significantly more likely to be pilfered compared to those of *C. henryi* (*χ* ^2^ = 8.409, df = 1, *p* < 0.01). Rodent species collectively imposed a significantly high risk of pilferage on both seed species (*χ* ^2^ = 36.794, df = 4, *p* < 0.001) (Figure 3A). Specifically, *L. edwardsi* exhibited the highest pilfering activity among the rodent species, followed by *A. chevrieri* , *N. fulvescens* , and *N. confucianus* (all pairwise comparisons, *p* < 0.01). Additionally, *A. chevrieri* showed significantly greater pilfering ability than *N. confucianus* (*p* < 0.05), and *A. draco* pilfered significantly more seeds than *N. fulvescens* (*p* < 0.01).

### 3.2 The Fates of Pilfered Seeds of *C. henryi* and *C. oleifera* by Rodent Species

Our results revealed a significant effect of rodent species on the re‐scatter‐hoarding probability of pilfered seeds (S‐SH: *χ* ^2^ = 32.454, df = 4, *p* < 0.001) (Figure 3B–F). Seed species did not significantly influence the re‐caching probability of pilfered seeds (S‐SH: *χ* ^2^ = 3.298, df = 4, *p* > 0.05). However, an interaction between rodent species and seed species was observed to significantly affect the re‐caching probability of pilfered seeds (S‐SH: *χ* ^2^ = 18.783, df = 4, *p* < 0.01). Scatter‐hoarding pilferers, such as *L. edwardsi* , re‐cached a significantly greater number of pilfered seeds compared to the other four rodent species (*p* < 0.001). Similar patterns were evident in the re‐larder‐hoarding probability of pilfered seeds (S‐LH: *χ* ^2^ = 23.778, df = 4, *p* < 0.001; *χ* ^2^ = 4.586, df = 4, *p* < 0.05; *χ* ^2^ = 24.545, df = 4, *p* < 0.001), and the number of pilfered seeds re‐cached by *L. edwardsi* differed significantly from that of the other four rodent species (S‐LH: *p* < 0.05). Additionally, we detected a significant effect of rodent species on the likelihood of pilfered seeds being consumed (S‐EAR: *χ* ^2^ = 20.16, df = 4, *p* < 0.001), where *L. edwardsi* tended to consume significantly more pilfered seeds than the other four rodent species (*p* < 0.05). Conversely, pilfered seeds of *C. oleifera* were more likely to be eaten in situ by pilfering rodents (S‐EIS: *χ* ^2^ = 44.155, df = 1, *p* < 0.001), with *A. draco* consuming significantly more pilfered seeds than the other four rodent species (S‐EIS: *χ* ^2^ = 44.033, df = 4, *p* < 0.001). Furthermore, pilfered seeds of *C. oleifera* were more likely to be abandoned by the pilfering rodents (S‐IAR: *χ* ^2^ = 9.176, df = 1, *p* = 0.002), with *A. chevrieri* abandoning significantly more pilfered seeds than the other four rodent species (S‐EIS: *χ* ^2^ = 17.874, df = 4, *p* = 0.001). The interaction effect between seed species and rodent species in re‐caching indicates that different rodent species adopt distinct caching strategies for the same type of seed.

## 4 Discussion

Our food hoarding experiments indicate that *L. edwardsi* primarily engage in scatter‐hoarding, with larder‐hoarding observed occasionally as a secondary behavior. Our findings demonstrate that five rodent species are capable of detecting seeds of *C. henryi* and *C. oleifera* that were previously concealed by *L.* *edwardsi*. Among the sympatric rodent species, there is a clear difference in their ability to pilfer scatter‐hoarded seeds from *L. edwardsi* . Notably, conspecific *L. edwardsi* exhibited a significantly higher ability to pilfer *C. henryi* seeds compared to other rodent species, whereas *A. draco* was more effective at pilfering *C. oleifera* seeds but less successful with *C. henryi* seeds. Alternatively, species that exhibited low pilfering rates may lack a natural inclination or preference for those specific seed species. This could be due to various factors such as the taste, texture, or nutritional content of the seeds. Prior research, however, has underscored that robust scatter‐hoarding behavior is intrinsically linked to heightened pilfering ability, as the proportion of cache pilfering correlates positively with scatter‐hoarding tendencies and inversely with larder‐hoarding inclinations across rodent species (Dittel et al. 2017; Wang et al. 2018). Our study unequivocally shows that *L. edwardsi, A. chevrieri*, and *A. draco* are skilled scatter‐hoarders with strong cache‐pilfering abilities, which are expected to be driven by their acute sense of smell and/or exploratory instincts. As prior studies confirm, scatter‐hoarders use precise spatial memory and strong olfactory abilities to locate and recover their caches (Vander Wall 2000; Briggs and Vander Wall 2004; Smulders et al. 2010; Steele et al. 2011). This extraordinary olfactory prowess not only aids them in retrieving their treasures but may also amplify their ability to discern and plunder caches belonging to fellow sympatric rodents (Vander Wall 2001).

Edward's long‐tailed rats ( *L. edwardsi* ) demonstrate the highest proficiency in scatter‐hoarding behavior locally, yet they pilfer fewer seeds of *C. oleifera* compared to *A. chevrieri* and *A. draco* . Our findings indicate that the capacity to pilfer scatter‐hoarded seeds is influenced not only by scatter‐hoarding tendencies but also by seed characteristics. Similarly, a previous study shows that *N. confucianus* exhibits a high rate of pilfering *Castanopsis hystrix* seeds while showing little interest in *Petrocosmea kerrii* seeds (Wang et al. 2014). These disparities can be attributed to differing preferences among rodent species for seeds with distinct traits. Additionally, our results indicate that rodents exhibiting scatter‐hoarding behavior ( *L. edwardsi* , *A. chevrieri* , and *A. draco* ) play a significant role in the secondary dispersal of *C. henryi* seeds. Furthermore, all rodent species except *A. chevrieri* were involved in the secondary dispersal of *C. oleifera* seeds, revealing interactions between rodents and specific plant species. Secondary dispersal by pilferers can extend the range over which seeds are spread, potentially increasing the likelihood of seeds reaching suitable germination sites. This extended dispersal can help plants colonize new areas, reduce competition with parent plants, and escape from density‐dependent mortality factors such as pathogens or predators. Secondary dispersal can also lead to a more heterogeneous distribution of seeds across the landscape, influencing the spatial structure of plant communities (Stapanian and Smith 1978; Hirsch et al. 2012). Cache pilfering and secondary dispersal processes have profound impacts on the spatial dynamics of plant populations and community ecology. The diversity of animal behaviors together with variation in seed traits jointly shape the mechanisms that maintain species coexistence and biodiversity within ecosystems, highlighting the critical role of cross‐species behavioral interactions in supporting ecosystem services and stability.

In our experiments, *A. draco* uniquely refrained from larder‐hoarding behavior following the pilfering of scatter‐hoarded seeds (including both *C. henryi* and *C. oleifera* ). Instead, they chose to either consume the pilfered *C. oleifera* seeds on‐site or transport *C. henryi* seeds to distant locations for subsequent consumption. This preference may stem from the relative ease of handling *C. oleifera* seeds in situ, whereas managing *C. henryi* seeds incurs significant time and energy costs. Furthermore, the relatively small body size of *A. draco* limits the effectiveness of larder‐hoarding as a strategy for protecting pilfered food. Notably, *L. edwardsi* and *A. chevrieri* avoided consuming *C. henryi* seeds on‐site, preferring instead to consume *C. oleifera* seeds in situ. Among the five rodent species examined, *L. edwardsi* demonstrated the highest larder‐hoarding rate for *C. henryi* seeds, whereas *N. confucianus* and *N. fulvescens* exhibited the highest larder‐hoarding rate for *C. oleifera* seeds. These findings align with the prior study, indicating that cache pilferers tend to larder‐hoard the seeds they pilfered from caches of scatter‐hoarders (Yang and Yi 2018). Seed size, energy costs, and behavioral trade‐offs related to animal body size reflect the adaptive strategies of rodents in resource utilization, influencing seed fate and plant population renewal strategies (Zhang et al. 2015). Caching and hoarding, as defensive strategies, can reduce the risk of repeated theft by other pilferers and enhance seed survival probability. Studying the trade‐offs between caching and scatter‐hoarding strategies under different environmental conditions and resource distributions helps to deepen the understanding of the ecological mechanisms by which animal population behaviors shape plant seed dispersal patterns, revealing the dynamic balance of interspecific coexistence and resource competition.

Despite being the predominant scatter‐hoarders, Edward's long‐tailed rats exhibit a marked preference for larder‐hoarding behavior after pilfering seeds, particularly those of *C. henryi* with their larger seed size—a strategy advantageous for safeguarding against secondary pilferage by other rodents. Previous studies have similarly documented variations in hoarding behavior when different seed species are encountered by a given rodent species (Chang and Zhang 2014; Wang et al. 2014; Zhang et al. 2016). These insights contribute to a deeper understanding of the mechanisms underlying the behavioral switch between scatter‐hoarding and larder‐hoarding in food‐hoarding rodents (Vander Wall et al. 2005; Dally et al. 2006). This study, using semi‐enclosure experiments, revealed differences in cache pilfering behavior among sympatric rodent species, thereby deepening our understanding of how animal behaviors shape seed fate and the complexity of ecological networks. However, numerous field factors, such as seed abundance, rodent density, cache density, soil water content, and cache defense behaviors, may influence cache pilfering behavior (Vander Wall 2000; Galvez et al. 2009; Lichti et al. 2017; Cao et al. 2018; Brzeziński et al. 2025). Only by examining specific behavioral patterns within multidimensional ecological contexts can we gain a deeper understanding of how animal hoarding behavior affects resource allocation, interspecific competition, and the dynamic balance of ecosystems.

Our study reveals that cache pilfering risks are influenced not only by seed traits but also by the identity of sympatric food‐hoarding rodent species. Caches established by scatter‐hoarding animals are more likely to be pilfered by conspecific scatter‐hoarders rather than larder‐hoarders, potentially because of the limited olfactory sensitivity or exploratory abilities of larder‐hoarding species (Vander Wall et al. 2003). Reciprocal cache pilferage among scatter‐hoarding animals may play a critical role in the redistribution of cached seeds, ultimately impacting seed survival and plant regeneration (Cao et al. 2018). Overall, the interplay between cache pilferage, seed traits, and rodent behavior represents a dynamic and complex relationship shaped by ecological and evolutionary pressures. Understanding this intricate relationship provides valuable insights into the ecological roles of rodents and the adaptive strategies employed by seeds in response to caching and pilferage behaviors. This dynamic interplay reflects the complex ecological and evolutionary interdependencies among animal behaviors, seed characteristics, and environmental pressures, illuminating the co‐evolutionary processes underlying animal–plant interactions. Gaining deeper insights into the feedback between cache pilferage and hoarding strategies can enhance our understanding of how species mediate resource competition and coexistence, ultimately influencing ecosystem functionality and resilience.

## Author Contributions

**Muha Cha:** data curation (equal), formal analysis (equal), writing – original draft (equal). **Yuan Li:** data curation (equal), formal analysis (equal), investigation (equal). **Minghui Wang:** conceptualization (equal), writing – original draft (equal), writing – review and editing (equal). **Xianfeng Yi:** conceptualization (equal), funding acquisition (equal), writing – original draft (equal), writing – review and editing (equal).

## Conflicts of Interest

The authors declare no conflicts of interest.

## Data Availability

Data generated from this work can be accessed at https://figshare.com/s/b3984a802e6a0325dad4.
